# Human Fibroblasts In Vitro Exposed to 2.45 GHz Continuous and Pulsed Wave Signals: Evaluation of Biological Effects with a Multimethodological Approach

**DOI:** 10.3390/ijms21197069

**Published:** 2020-09-25

**Authors:** Elisa Regalbuto, Anna Anselmo, Stefania De Sanctis, Valeria Franchini, Florigio Lista, Monica Benvenuto, Roberto Bei, Laura Masuelli, Guglielmo D’Inzeo, Alessandra Paffi, Eugenio Trodella, Antonella Sgura

**Affiliations:** 1 Scientific Department, Army Medical Center of Rome, 00184 Rome, Italy; annanselm@gmail.com (A.A.); stefania.desanctis@gmail.com (S.D.S.); valeriafrn@gmail.com (V.F.); romano.lista@gmail.com (F.L.); 2 Department of Science, University of Rome “Roma Tre”, 00146 Rome, Italy; 3 Saint Camillus International University of Health and Medical Sciences, 00131 Rome, Italy; monicab4@hotmail.it; 4 Department of Clinical Sciences and Translational Medicine, University of Rome “Tor Vergata”, 00133 Rome, Italy; bei@med.uniroma2.it; 5 Department of Experimental Medicine, University of Rome “Sapienza”, 00161 Rome, Italy; laura.masuelli@uniroma1.it; 6 Department of Information Engineering, Electronics and Telecommunications (DIET), University of Rome “La Sapienza”, 00184 Rome, Italy; guglielmo.dinzeo@uniroma1.it (G.D.); alessandra.paffi@uniroma1.it (A.P.); eugeniotrodella@gmail.com (E.T.)

**Keywords:** 2.45 GHz, Wi-Fi, genotoxic effect, gene expression, RNA sequencing (RNA-seq), RT-PCR

## Abstract

The increasing exposure to radiofrequency electromagnetic fields (RF-EMF), especially from wireless communication devices, raises questions about their possible adverse health effects. So far, several in vitro studies evaluating RF-EMF genotoxic and cytotoxic non-thermal effects have reported contradictory results that could be mainly due to inadequate experimental design and lack of well-characterized exposure systems and conditions. Moreover, a topic poorly investigated is related to signal modulation induced by electromagnetic fields. The aim of this study was to perform an analysis of the potential non-thermal biological effects induced by 2.45 GHz exposures through a characterized exposure system and a multimethodological approach. Human fibroblasts were exposed to continuous (CW) and pulsed (PW) signals for 2 h in a wire patch cell-based exposure system at the specific absorption rate (SAR) of 0.7 W/kg. The evaluation of the potential biological effects was carried out through a multimethodological approach, including classical biological markers (genotoxic, cell cycle, and ultrastructural) and the evaluation of gene expression profile through the powerful high-throughput next generation sequencing (NGS) RNA sequencing (RNA-seq) approach. Our results suggest that 2.45 GHz radiofrequency fields did not induce significant biological effects at a cellular or molecular level for the evaluated exposure parameters and conditions.

## 1. Introduction

Radiofrequency electromagnetic fields (RF-EMF) include frequencies ranging from 3 kHz to 300 GHz belonging to the non-ionizing part of the electromagnetic spectrum. Different devices emitting in this range of frequency are currently used in industry, medicine, military, and telecommunication applications.

An exponential increase in the adoption of RF-EMF in wireless communication technologies, such as Wireless Fidelity (WiFi, Bluetooth, etc.), occurred in the last few decades. Therefore, the continuous human exposure to RFs within living and working environments rises concerns about their potential biological effects and possible associated health risks.

Currently, safety standards for limiting human exposures to RF-EMF are based on the well-characterized thermal effect [1]; however, since the amount of energy involved in chronic or long-term exposures to the low-level RF-EMF would not significantly increase the temperature of a cell, tissue, or organism, the understanding of potential non-thermal RF effects has been the focus of several studies.

Although some hypotheses about the non-thermal mechanism of interaction between RF and biological tissue have been proposed [2,3,4], results from the scientific literature are often contradictory and there are still more open questions available than answers.

Therefore, more research is needed, following a multiscale methodology, starting from a first transduction step at molecular scale [4].

Moreover, an interesting scientific question is related to possible different biological effects induced by continuous and modulated wave signals, which occur in a wide variety of RF applications (radar, wireless communications, broadcast communications, and industrial processes). To date, this topic is poorly investigated, and no clear results are reported [5].

Since the effect of RF radiation significantly depends on many variables (frequency, dose rate, waveform, exposure time, modulation, temperature, exposure condition, cellular type, endpoints), the controversial findings can be often related to an inadequate experimental design and a lack of well-characterized exposure systems and conditions [6,7]. In order to ensure non-thermal exposure levels and provide a correct estimate of the specific absorption rate (SAR), much care must be placed in dosimetry, which is the basic requirement for all high-quality investigations [8,9,10]. 

Many in vitro studies focus on a possible RF genotoxic effect exerted on DNA molecules, such as single strand breaks (SSBs) and double strand breaks (DSBs) and/or chromosomes, causing structural or numerical aberrations in cells that are crucial events in the carcinogenesis process [11]. Based on the limited evidence of the carcinogenic potential in both human and animal investigations, RF exposure is classified as a possible human carcinogen (class 2B) [12]. 

Most of the studies assessed DNA SSBs and DSBs through comet assay, reporting, in many cases, no DNA damage [13,14]. A more specific and sensitive method to evaluate the presence of DSBs based on the identification of the phosphorylated H2AX histone (γ-H2AX), alone or in association with p53 binding protein 1 (53BP1), was used in a few studies. Some of these researches, performed on different cellular models exposed to various RF fields, reported no induction of DSBs [15,16]. Interestingly, some studies reported a significant reduction in the number of γ-H2AX/53BP1 in human lymphocytes from both healthy and hypersensitive persons exposed to different frequencies (915 MHz, 905 MHz), suggesting that this frequency could affect cells in a manner similar to the stress factor [17,18].

The most common method used to evaluate the chromosomal damage is the micronucleus assay, in which the presence of micronuclei (MN), which may originate from chromosome fragments or whole chromosomes, are assessed in binucleated (BN) cells [19]. Most of the studies employing this endpoint have been performed on human blood cultures and showed contradictory results, reporting an increased MN induction in the cultures exposed to different frequency ranges [20,21,22,23,24,25,26] or no effect [23,27,28,29,30,31,32].

In order to identify the different MN origins, which may reflect structural as well as numerical chromosome aberrations, some authors combined the results obtained from the MN analysis and the comet assay. Among these studies, no DNA damage or MN induction was observed in several cellular models exposed with different parameters and conditions [29,30,33]. Conversely, Tice et al. observed an increased MN frequency in lymphocytes, following 24 h of exposure (835 MHz, SAR 10 W/kg), and an absence of DNA damage by comet assay, thus suggesting an aneugenic effect [24]. Another strategy, which allows the discrimination of MN arising from entire chromosomes from those arising from acentric fragments, is based on the presence or absence of centromere in MN through the evaluation of a fluorescent signal. Schwarz et al. performed fluorescence in situ hybridization (FISH) for all centromeres and reported an increased DNA damage in human fibroblast, but not in lymphocytes, exposed to 1.9 GHz (CW; SAR = from 0.05 to 2 W/kg) in a dose and time-dependent way due to increased MN frequency based on the formation of acentric fragments and an enhanced comet tail factor [34].

Some authors evaluated aneuploidy events using a FISH-based approach for a specific chromosome pair in interphase cells. Among these, Mashevich et al. observed an increased aneuploidy for chromosome 17 on human peripheral blood lymphocytes, exposed for 72 h to 830 MHz as a function of the SAR (CW; SAR = from 1.6 and 8.8 W/kg) [35]. This finding was confirmed in a later study performed on the same cellular model and exposure conditions, in which an increased rate of aneuploidy was observed for chromosome 1 and 10 (SAR = 4.1 W/kg) and for chromosome 11 and 17 (SAR = 2.9 W/kg) [36].

Only in one study, FISH was performed in metaphase human peripheral blood lymphocytes exposed to 1.9 GHz for 24 h (SAR = 0.5 and 2 W/kg) and it was reported that SAR may either influence the repair of X-ray-induced DNA breaks or alter the cell death pathways of the damage response [37].

The different MN origin can be also evaluated by using centromeric labeled antibodies (CREST analysis), whereas centromere-negative MN (MN−) reflects chromosome breakage and centromere-positive MN (MN+) indicates chromosome loss. In a recent study, Franchini et al. reported aneuploidy induction in human fibroblasts exposed to 25 GHz (SAR = 20 mW/g), due to chromosome loss highlighted by a significant increase in MN+ assessed by MN-CREST analysis on interphase cells [16].

On the contrary, Bourthoumieu et al. found no significant change in the rate of aneuploidy using interphase FISH for chromosomes 11 and 17 in human amniotic cells exposed for 24 h to 900 MHz (SAR = 0.25, 1, 2 and 4 W/kg) [38].

Other extensively evaluated endpoints are related to cell proliferation and cell cycle analysis. Few in vitro studies reported changes in cell proliferation rate [39,40] or an impaired cell cycle after RF exposure [41,42], whereas most studies reported no effect [16,31,43,44,45,46,47].

Very few investigations focused on the identification of cellular morphological changes through ultrastructural observation. Erdine et al. described different degrees of damage in mitochondria, microtubules, and microfilament of rat’s axon exposed to the pulsed RF field [48], whereas Franchini et al. reported a normal cellular structure exhibited by human fibroblast after exposure to 25 GHz [16].

In order to understand the underlying mechanism between RF and the biological matter, a new interesting field of research is related to the identification of possible sensitive genes, which change their expression profile after exposure. To date, few large-scale studies performed using microarray technology have investigated RF-EMF effects on gene expression with contradictory results, showing that it is difficult to find a marked cellular response to this radiation.

Zhao et al. reported that intermittent in vitro exposure of rat neuron cultures to 1.8 GHz increases or decreases the expression of genes involved in multiple cellular functions, including cytoskeleton, signal transduction pathways, and metabolism [49].

Some authors, who exposed different human cell types to 900 and 1800 MHz, reported a cell-dependent effect of RF exposure on gene expression [50,51].

Le Quèment et al. showed that 60 GHz had no massive effect on human keratinocytes but could change the expression levels of some genes [52]. In a subsequent study, performed on the same cellular model and frequency, whole transcriptome analysis revealed a slight but specific radiation effect on gene expression in hyperthermia conditions [53]. However, in several other studies, no effect on gene expression was reported on different cellular models exposed to various RF fields using microarrays [54,55,56,57,58,59].

Among the in vivo studies, Dasdag and co-authors reported that chronic exposure to 2.45 GHz increased the expression of some microRNAs (miRNA) in rat brains [60]. In another in vivo study, McNamee et al. evaluated gene expression changes in several rodent brain regions exposed to 1.9 GHz (CW and PW RF fields; 4 h/day for 5 consecutive days), reporting no consistent changes in gene expression [61].

The introduction of high-throughput technologies, such as next generation sequencing (NGS), which also allows whole transcriptome analysis through RNA sequencing (RNA-seq), seems to be a powerful and promising approach to identify RF-EMF specific gene expression patterns.

To the best of our knowledge, only three in vivo studies evaluated the genome-wide mRNA expression profile using the Illumina sequencing technology, reporting differentially expressed genes in *Caernorhabditis elegans* after prolonged exposure to 1.75 GHz (SAR = 3 W/kg) [62] and in some genes related to energy metabolism in budding yeast after exposure to 50 Hz and 2.0 GHz [63]. A more recent study performed on *Escherichia coli* K-12 DH5α exposed to 2.4 GHz for 5 h reported that most of the genes identified were involved in many cellular and metabolic processes [64].

In this heterogeneous context, the present in vitro study focused on the biological effects of 2.45 GHz at a SAR value of 0.7 W/kg through a well-characterized exposure system and condition. Moreover, both continuous (CW) and pulsed (PW) wave signals were evaluated, since, to date, few and controversial results are available on this topic.

The aim of this study is to provide a comprehensive insight into the potential RF biological effects at the cellular level through a multimethodological approach that includes genotoxic, cell cycle, and morphological analyses and genome-wide mRNA expression profile using a high-throughput sequencing approach (RNA-seq), attempting to elucidate the underlying mechanism.

## 2. Results

The overall results of this study suggest that 2.45 GHz doesn’t induce genotoxic and cytotoxic effects with both CW and PW types of signals after 2 h of exposure at the SAR of 0.7 W/kg. Interestingly, the results obtained from gene expression analysis, performed through the NGS approach (RNA-seq) at different time points after exposure (0 min, 2 and 24 h), showed a modulation of some genes involved in multiple functions. Although not all the genes selected for RT-PCR validation were confirmed as significantly differentially expressed and showed a different fold change values, a similar trend in the direction of change observed by RNA-seq was confirmed.

### 2.1. Cell Cycle Analysis 

The FACS analysis on DNA content was performed 2 and 24 h after exposure to 2.45 GHz (CW or PW). Results obtained from the cells exposed to the two different types of signals were compared to those of the corresponding sham samples. No significant changes in the different phases of the cell cycle were observed 2 and 24 h after exposure to both CW and PW signals (Table 1).

### 2.2. γ-H2AX/53BP1 Assay

**Figure 1:**
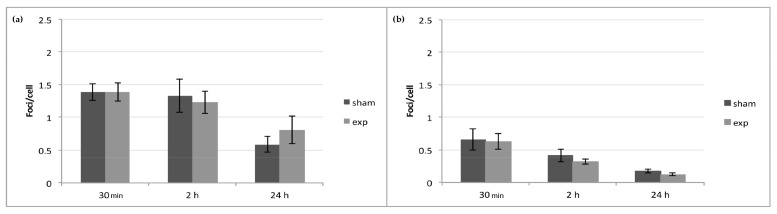
H2AX histone/p53 binding protein 1 (γ-H2AX/53BP1) foci analysis on HDF exposed to 2.45 GHz. No significant differences were observed 30 min, 2 h, or 24 h after exposure to 2.45 GHz CW (**a**) and PW (**b**), with respect to sham samples. Results obtained with the cells exposed to CW or PW signals were compared to those obtained with the corresponding sham cells (exposed 30 min vs. sham 30 min; exposed 2 h vs. sham 2 h; exposed 24 h vs. sham 24 h; 2-tailed *t*-test). Data are representative of four experiments, and bars denote the standard error.

The analysis of colocalized γ-H2AX/53BP1 foci showed no significant differences (*p* > 0.05) between exposed and sham samples (exposed 2 h vs. sham 2 h; exposed 24 h vs. sham 24 h) for both types of signals (CW or PW) (Figure 1).

### 2.3. Micronuclei Anti-Kinetochore Antibody (CREST) Analysis

**Figure 2:**
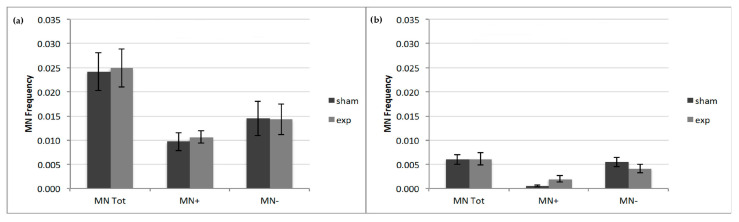
CREST analysis on HDF exposed to 2.45 GHz. No significant increase in MN tot or in the frequency of MN+ and MN− in 2.45 GHz CW (**a**) and PW (**b**) exposed, with respect to sham samples were observed. Data are representative of four experiments, and bars denote the standard error.

For both CW and PW signals, CREST analysis on exposed samples, with respect to the sham samples, showed no significant increase in the total number of MN, corresponding to the sum of CREST positive (MN+) and CREST negative (MN−) micronuclei. Similarly, no significant increase was observed in the frequency of MN+ and MN− in the exposed samples (CW or PW), with respect to the sham samples (Figure 2).

### 2.4. Ultrastructural Analysis

**Figure 3:**
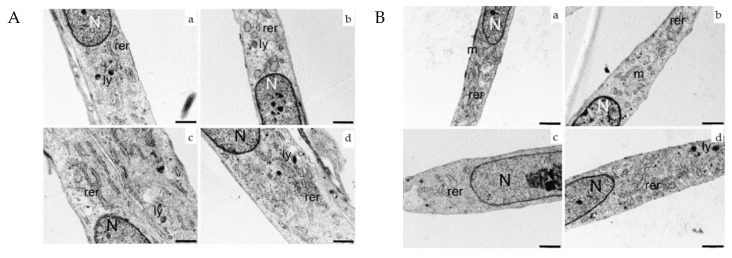
Ultrastructural analysis of HDF cells exposed to 2.45 GHz. (**Panel A**): sham (**a**,**c**) and 2.45 GHz CW- (**b**,**d**) exposed HDF. Exposed cells were examined 2 h (**b**) and 24 h (**d**) after exposure. Nucleus (N), rough endoplasmic reticulum (rer), lysosome (ly), mitochondria (m) (bars correspond to 1 µm). No morphological differences were found between sham or exposed cells. (**Panel B**): sham (**a**,**c**) and 2.45 GHz PW- (**b**,**d**) exposed HDF. Exposed cells were examined 2 h (**b**) and 24 h (**d**) after exposure. (Bars correspond to 1 µm). No morphological differences were found between sham and exposed cells.

Ultrastructural analysis was performed on HDF cells exposed to 2.45 GHz (CW or PW) and, compared to sham control cells, 2 and 24 h after exposure. No morphological differences were observed between sham and CW or PW exposed cells, as shown in Figure 3. Sham and exposed samples from both signals (CW or PW) and for each time point evaluated appeared as elongated cells with elongated centrally located nuclei, essentially formed by euchromatin with poor heterochromatin and well-organized nucleoli. Abundant rough endoplasmic reticulum sometimes dilated, and few mitochondria and lysosomes were visible in the cytoplasm. 

### 2.5. Gene Expression Profiling 

Gene expression profiling was evaluated using the mRNA-seq approach on the Illumina NextSeq 500 platform. The analysis was performed on RNA samples isolated from sham and 2.45 GHz (CW or PW) exposed cells at three different time-points: immediately after and 2 and 24 h post-exposure (0 min; 2 h; 24 h). For both CW and PW signals, four experimental replicates were performed, with a total of 48 sequenced samples (24 for CW experiment and 24 for PW experiment). For each run, eight samples were sequenced, with an average of 50 million generated reads per sample and about 8% of poor-quality trimmed reads.

To identify genes presenting significant changes in expression, two different statistical tools were used (DESeq2 and EdgeR). The thresholds of false discovery rate (FDR) < 0.01 were used to determine the significantly up-regulated or down-regulated genes between different groups. Thereafter, the above statistical analysis, performed with DESeq2 and EdgeR, was repeated under lower-stringency parameters, without FDR-adjustment, considering a *p*-value ≤ 0.05 and a fold change of 1.5 as the minimum cut-off value. 

#### 2.5.1. Differential Gene Expression Analysis 

Differential gene expression (DGE) between cells exposed to 2.45 GHz (CW and PW) and sham-control samples, following statistical analysis using an FDR-adjusted *p*-value cut-off, evidenced only one target gene in the samples examined 2 h after CW exposure. This probe was a long non-coding RNA (RNA Component of Mitochondrial RNA Processing Endoribonuclease (RMRP) that resulted in down-regulation. 

The statistical analysis was also performed without the FDR correction, considering *p* ≤ 0.05 and a fold change of 1.5 as the minimum cut-off value. With this approach, a total of 53 genes, for CW exposed samples, and 33 genes, for PW exposed samples, were identified as differentially expressed in exposed samples (Table 2 and Table 3). Interestingly, 6 (5 up-regulated; 1 down-regulated) and 5 genes (3 up-regulated; 2 down-regulated) were differentially modulated in the samples processed immediately after exposure to 2.45 GHz CW and 2.45 GHz PW, respectively. In the samples analyzed 2 h after CW and PW exposure, 39 (19 up-regulated; 20 down-regulated) and 22 genes (9 up-regulated; 13 down-regulated) were identified, respectively. On the other hand, only 8 (5 up-regulated; 3 down-regulated) and 7 genes (up-regulated) were found modified, 24 h after exposure to CW and PW, respectively. For both types of signals evaluated, most of the responsive genes were identified 2 h after exposure, thus suggesting a transient and reversible cellular response. Only 3 genes (*RN7L1*, RNA, 7SL, cytoplasmic 1; *RN7L2*, RNA, 7SL, cytoplasmic 2; *ANKRD36C*, Ankyrin repeat domain-containing protein 36) were observed to be down-regulated (fold change > 1.5), both 2 and 24 h after exposure to 2.45 GHz CW, with respect to the sham group. Only 2 genes (RMRP, RNA Component of Mitochondrial RNA Processing Endoribonuclease; *AHNAK2*, Protein AHNAK2) showed an altered gene expression profile 2 h after exposure to both CW and PW exposures.

#### 2.5.2. DGE Functional Classification

**Figure 4:**
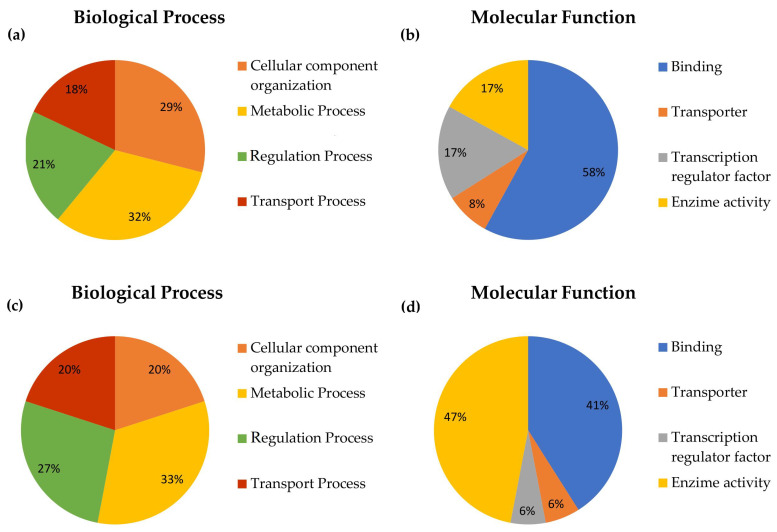
Percentage of differential gene expressions (DGEs) involved in the different biological processes and molecular functions, as reported in gene ontology (GO) term analysis, for both types of signals evaluated. (**a**) Percentage of genes involved in each biological process after exposure to 2.45 GHz CW. (**b**) Percentage of genes involved in each molecular function after exposure to 2.45 GHz CW. (**c**) Percentage of genes involved in each biological process after exposure to 2.45 GHz PW. (**d**) Percentage of genes involved in each molecular function after exposure to 2.45 GHz PW.

To determine whether CW or PW exposure could affect the pattern of gene expression, gene ontology (GO) terms analysis of the DGEs was performed on three ontology levels: biological processes, molecular functions, and pathways. The results are summarized in Figure 4.

**Figure 5:**
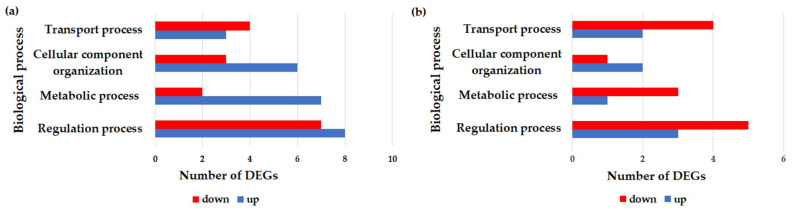
GO functional networks for down-regulated and up-regulated genes for 2.45 CW exposed samples (**a**) and 2.45 PW exposed samples (**b**). The down- and up-regulated genes are shown in red and blue, respectively.

For the samples exposed to 2.45 GHz CW, the modulated genes results, mainly involved in metabolic and cellular component organization (Figure 4a) and their most relevant molecular function, were related to binding activities (Figure 4b). No significantly-affected signaling transduction pathways were identified. The results obtained for the genes differentially expressed after exposure to 2.45 GHz PW indicated that the most relevant biological processes in which they were involved were related to metabolic or regulation processes (Figure 4c), with many of them having binding or catalytic activity (Figure 4d). Even for this type of exposure, no significantly-affected signaling transduction pathways were identified. Additional information about the up- and down-regulated genes within each functional network for the two type of signals are reported in Figure 5.

#### 2.5.3. Quantitative RT-PCR Validation

In order to ensure the reproducibility of the results, RT-PCR validation was performed on five genes (*RMRP,* RNA Component Of Mitochondrial RNA Processing Endoribonuclease; *Alms1*, Alstrom syndrome protein 1; *BSN,* Protein bassoon; *SYNE2*, Nesprin-2; *AHNAK2,* Protein AHNAK2), selected on the basis of their fold change and *p*-value. The RT-PCR validation step was performed on the same RNA used for the RNA-seq, derived from four independent exposures.

RT-PCR analysis showed that only one gene, among the five selected, could be confirmed as a differentially expressed gene (Table 4). This gene was *BSN* (*t*-test, *p* = 0.0097), which encodes for a scaffolding protein that appears to be involved in the organization of the presynaptic cytoskeleton. Although the fold change of the selected genes detected by RT-PCR were not the same as those detected by RNA-seq, the results agreed in terms of direction of change, as reported in Table 4.

## 3. Discussion

The goal of the current study was to provide a complete insight into the potential RF biological effects at the cellular and molecular level, in human fibroblasts in vitro exposed to 2.45 GHz through a multimethodological approach and well-characterized exposure systems and conditions. 

The selected frequency was 2.45 GHz, because the widespread use of Wi-Fi technologies in everyday life is leading to concerns about possible health consequences. Moreover, because of the growing interest in possible different biological effects related to signal modulation, this research focused on the study of both CW and PW signals, with a SAR value of 0.7 W/kg, below the maximum limit of 2 W/kg recommended by the European guidelines for limiting the exposures to RF-EMF [1]. The used dose is considered the worst case, since, in actual everyday exposure to Wi-Fi signals, this level is hardly achievable due to the low radiated power (100 mW EIRP) imposed by the international standards [65] for this kind of technology.

Many investigations assessed the non-thermal biological effects, especially the genotoxic potential, of these radiations through in vitro and in vivo studies [13,66]. Although some hypothesis about the possible non-thermal mechanism of interactions have been proposed, very few of them can explain these controversial outcomes [2,3,4]. The difficulties in the interpretation of the results could be related, in some cases, to temperature increase during the experiment, inadequate experimental procedure, or inexhaustive information provided. In the light of these observations, in the present study, much emphasis has been placed on the exposure system and a well-considered experimental design. In order to exclude that any biological alterations associated with the exposures could be related to thermal effects, during the experiments cell culture temperature was monitored through a Fluoroptic Thermometer. The temperature inside the Petri dishes remained at 37 ± 0.25 °C during the 2 h of exposure. Accordingly, the reported results are of a non-thermal nature. Moreover, in order to minimize the individual biases, all experiments for each endpoint assessed were performed at least in triplicate and the analyses were carried out in a blind manner [10,67].

The analysis of possible non-thermal genotoxic and cytotoxic effects suggests that 2.45 GHz did not induce neither aneugenic or clastogenic effects within the exposure conditions evaluated and there were no significant differences between the two types of signals tested. Our findings regarding flow cytometry analysis are in accordance with previous studies investigating the effects of different RF radiation on various cellular types [16,68,69,70], showing no effects on the cell cycle. The evaluation of chromosomal damage and its origin, assessed by indirect immunofluorescence CREST-MN analysis, showed no aneugenic or clastogenic effects for both types of signals. These observations are in agreement with other studies evaluating chromosomal damage by the conventional MN assay on different cellular types exposed to 2.45 GHz [71,72] or to other RF ranges [30,73,74]. Moreover, the absence of a clastogenic effect was confirmed by the γ-H2AX/53BP1 foci assay, which showed no significant induction of DSBs. This finding was in agreement with a previous study, in which γ-H2AX/53BP1 was assessed on the same cellular type exposed to a different RF radiation [16]. Additionally, other investigations confirm this result by using comet assay on human lymphocytes [73] or on different cellular types exposed to Wi-Fi frequencies with both continuous and modulated signals [75].

No morphological changes related to 2.45 GHz exposures for both signals and time-points evaluated were observed by transmission electron microscopy, according to previous in vitro studies on human dermal fibroblasts [16] or on other cellular models [76] exposed to different types of RF radiation.

The current study is relevant, with regard to gene expression analysis. To the best of our knowledge, this is the first study assessing gene expression alterations in human cells in vitro exposed to RF using the high-throughput RNA-seq approach.

The mRNA sequencing was performed at three different time points (0 min, 2 h, and 24 h) after exposure to both CW and PW 2.45 GHz, and, in order to give consistency and robustness to the results, four experimental replicates were performed. The identification of differentially expressed genes, performed under rigorous statistical analysis (FDR), showed only one down-regulated long non-coding RNA (RMRP, RNA Component of Mitochondrial RNA Processing Endoribonuclease) 2 h after 2.45 GHz CW exposure. 

Since the application of FDR analysis may have determined the exclusion of some true positive events (type 2 error), a second statistical analysis was performed without FDR-corrected approach [61,77], by which only those genes with *p*-values less than 0.05 and a fold-change of 1.5 were selected. Using this method, as expected, some genes showed differential expression profiles for both types of exposures and for each time point evaluated. After gene ontology terms analysis, these genes resulted involved in multiple biological processes related to metabolism, signal transduction, and cellular component organization. Most of the transcripts, belonging to the same functional class, were either up- or down-regulated, making it difficult to establish a common trend. Overall, the results indicated that, for both types of signals, there is a minimal induction of genes with altered expression profile immediately after exposure and the number increased 2 h post-exposure, while few genes were detected 24 h after exposures. Furthermore, the genes appear to follow a time-dependent modulation profile, since they showed distinct temporal response. There were no differentially expressed genes in common between the two types of signals, other than RMRP, which showed a down-regulated expression profile 2 h after both CW and PW exposures, and *AHNAK2* (Protein AHNAK2), for which the pattern of expression was the opposite with the different types of exposure (up-regulated 2 h after CW exposure and down-regulated 2 h after PW exposure). 

Since differences detected by mRNA-seq technology after RF-EMF exposure may result in very small changes, in order to exclude false positive results, five genes were selected for RT-PCR validation, based on fold change, low *p*-value, and biological function. These include the long non-coding RNA RMRP, which showed a down-regulated expression profile 2 h after exposure to both CW and PW signal and was also the only expression profile detected under FDR statistical analysis (after CW exposure). Although RMRP functions are not fully understood, it has been reported that it may play a role in mitochondrial DNA replication, where it cleaves the RNA primer, consisting of an RNA/DNA hybrid, which initiates the mitochondrial DNA replication [78]. Moreover, RMRP seems to form, together with the catalytic subunit of the telomerase (hTERT), an RNA-dependent RNA polymerase that converts single-stranded RMRP RNA into double-stranded RMRP [79]. Another selected gene was *AHNAK2,* differentially expressed 2 h after both CW and PW exposure, with an opposite direction of change. This gene encodes for a secreted protein that appears to be implicated in metabolic processes that are involved in signal transduction mechanisms [80]. Finally, some up-regulated cytoskeleton-related genes, identified 2 h after exposure to CW, including *ALMS1* (Alstrom syndrome protein 1), *BSN* (Protein bassoon), and *SYNE2* (Nesprin-2), were selected for the validation. These genes seem to be interesting, since previous investigations speculated that RF might cause perturbation on the cytoskeleton and spindle assembly through microtubules vibration [81] and some studies suggested aneuploidy induction after exposure to RF [16,35,36], which could be related to problems in spindle microtubule formation, resulting in defects in the attachment to kinetochores. 

RT-PCR validation analysis showed that, among the selected genes, only *BSN* could be confirmed as differentially expressed (*t*-test, *p* = 0.0097). This gene, primarily expressed in neurons in the brain, encodes for a scaffolding protein that seems to be involved in the organization of the cytoskeleton in a specific site in the axon terminal, which regulates neurotransmitter release (https://www.ncbi.nlm.nih.gov/gene/8927#gene-expression). It also participates in the formation of vesicles that are transported along axons to sites of synaptic contacts. In addition, *BSN* was also found to regulate Wnt signaling pathway (involved in signal transduction pathway) [82]. Even if transient, the overexpression of this cytoskeleton-related gene in dermal fibroblasts after RF exposure is of interest, since some previous studies suggested the ability of RF to influence cytoskeleton structure [16,35,36,81]. The role of this gene in regulating Wnt is noteworthy because this signaling pathway is involved in skin development, cell migration, and cell polarity [83], and an alteration of this has been reported to be a causative factor for a number of pathologies, including breast, colon, and skin cancer [84,85]. Additional studies in dermal cells should be carried out to understand biological function of *BSN* in this tissue and its possible involvement in the biological response to RF exposure. 

In the present study, despite the fold change detected by mRNA-seq, which was not the same and was generally larger than that obtained by RT-PCR, as reported in a previous study [63], it was possible to observe, for all the genes analyzed, a similar trend between the results obtained from the two methodologies, in terms of direction of change. The discrepancies between the fold changes observed with the two methods can be related to the differences in the normalization process of data analysis between the two techniques, since the unit of measure is different. For this reason, we can suppose that the missing confirmation through validation for most of the genes can be related to the subtle nature of changes and to the methodological differences between mRNA-seq and RT-PCR. This suggests the usefulness of using both techniques in order to obtain an unambiguous result.

Considering previous studies evaluating the effect of Wi-Fi radiation on gene expression, some in vivo studies reported that long-term exposure to 2.4 GHz altered the expression of miRNA and gene expression in rat brains [60,86]. A recent study, investigating the alterations in the bacterial transcriptome profiling, reported an influence on genes responsible for metabolic and cellular processes, localization, stress response, transposition, motility, chemotaxis, and cell adhesion after exposure to Wi-Fi radiofrequency radiation [64].

Among the in vitro studies, Sakuraj et al. reported no significant gene expression modulation in human glial cells at different SAR values (1, 5, and 10 W/kg) and times of exposure (1 h, 2 h, and 24 h) using DNA microarray [56]. Another research, performed on HL-60 cells exposed for 2 or 6 h (SAR 10 W/kg), reported several genes differentially expressed, but these results originate from a single experiment and were not confirmed by RT-PCR [87].

The comparison between our results and previous studies evaluating the RF-induced gene modulation is difficult because they are still very heterogeneous. This variability could be related to different frequencies, biological models, and methodological approaches used. Some of the studies reporting positive findings are based on an insufficient number of experimental replicates and suffer methodological limitation, due to the lack of validation through RT-PCR [50,87,88]. The strength of our study is related to the use of a very rigorous multimethodological approach, due to the high number of replicates, the controlled experimental conditions, especially for the exposure system, and the use of the high-throughput RNA-seq analysis in combination with the RT-PCR validation.

In summary, the present work suggests that, within the exposure conditions evaluated, 2.45 GHz did not induce non-thermal genotoxic effects. With regard to gene expression analysis, the present study provides the most comprehensive analysis of potential gene expression changes in human fibroblasts exposed to Wi-Fi. No evidence of altered gene expression was observed under FDR-corrected analysis, whereas some genes differentially expressed were identified using a non-FDR statistical analysis. Most of these genes were involved in biological processes, such as the metabolism, signal transduction, and cellular component organization. Interestingly, for both types of signals evaluated, most of the responsive genes were identified 2 h after exposure, thus suggesting a transient and reversible cellular response.

Concluding, the approach proposed in this study may open the door to additional high-quality investigations, since the improvement of experimental quality, through appropriate procedural protocols, is crucial in this research field in order to address more clearly the often-ambiguous results on the potential adverse effects of RF-EMF exposure on human health. Moreover, further studies employing RNA-seq analysis on different cellular models and for longer times of exposure are recommended in order to provide additional insight regarding the mechanism of possible Wi-Fi effects on biological systems and also to give a contribution to ensure the validity of the results in terms of robustness, accuracy, and reproducibility.

## 4. Materials and Methods 

### 4.1. Exposure System and Dosimetry

**Figure 6:**
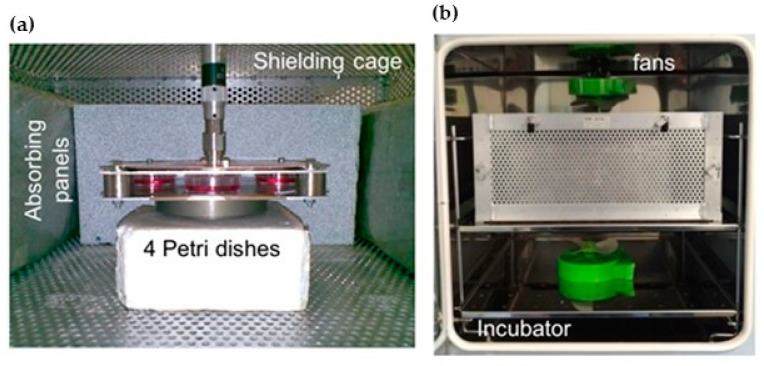
Picture of the WPC with the four Petri dishes inside. (**a**) WPC placed into the shielding cage with the lateral walls covered with absorbing panels. (**b**) Shielding cage inside the incubator with the two fans used to induce a forced air flow for temperature control.

To carry out the investigation, the exposure system published in [89] was used. It is based on a WPC suitably designed to operate at the Wi-Fi frequencies (2.40–2.48 GHz). As discussed in [89], a WPC is a special kind of resonant EM structure, where the biological samples are placed between two squared metallic plates short-circuited at each corner. This kind of system is generally compact and presents quite good efficiency values in terms of induced SAR in the sample per unitary input power. Being an open structure, it guarantees an easy accessibility to the sample but needs an EM-compatible arrangement. Due to the small space around the samples, generally, a mechanism for the local temperature control is also needed. The WPC used in this work can allocate four 35 mm Petri dishes, filled with 2 mL of biological samples in correspondence of each side, and is small enough (250 mm side, 15 mm height) to fit inside a commercial incubator, as shown in Figure 6. The efficiency, calculated by means of numerical simulations and experimentally validated in [89], is equal to 1.1 (W/kg)/W in cell monolayers, with a homogeneity of 70%.

To avoid EM interference with the incubator, the WPC was placed inside a perforated metal cage and internally covered with foam absorbing panels (Figure 6a).

The samples were exposed for 2 h either to a CW at 2.45 GHz or to the same carrier modulated in amplitude with a square pulse 1 ms period and 50% duty cycle (PW), representative of a Wi-Fi burst. The signal was delivered to the WPC by a generation system and remotely controlled by a LabVIEW™ program, which can generate both CW and differently modulated signals [90,91].

The chosen dose was 0.7 W/kg, which was below the limit of 2 W/kg set by the international regulations (ICNIRP). In actual everyday human exposure to Wi-Fi signals, this level is hardly achievable due to the low radiated power (100 mW EIRP) imposed by the international standards for this kind of technology [64]. Therefore, the used dose is considered as the worst case. In the meantime, this dose guarantees the avoidance of possible confounding thermal effects when a simple setup for temperature control, based on two fans (see Figure 6b), is used. Details on temperature measurements are given in Section 4.2.

Considering the WPC efficiency, to obtain a SAR of 0.7 W/kg, averaged over time, inside the cell monolayer, the incident power was set to 0.6 W and 1.2 W for the CW and the PW, respectively.

During the experiments, the incident power was continuously monitored using a bi-directional coupler and an Agilent E4419B Power Meter.

### 4.2. Temperature Monitoring

One of the aspects that must be accurately monitored and controlled during the exposure to EM fields is the temperature increase, in order to prevent thermal effects to mask possible non-thermal effects [67].

**Figure 7:**
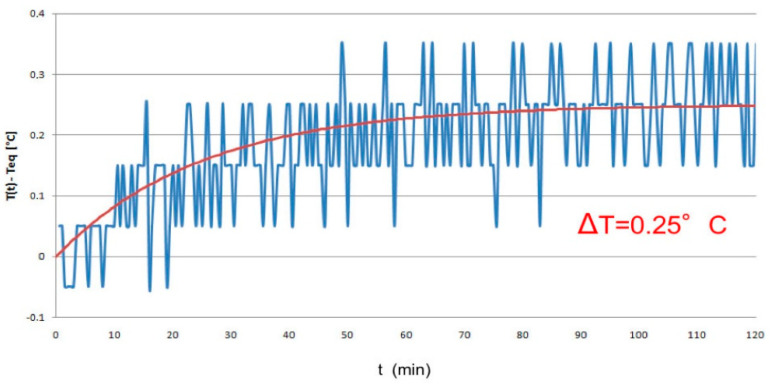
Temperature increase as a function of time. Measurement inside the biological sample during 2 h exposure to a CW at 2.45 GHz and 0.7 W/kg of SAR.

Temperature was measured inside one of the Petri dishes filled with 2 mL of culture medium using a Luxtron 712 Fluoroptic probe during the exposure to the CW at 2.45 GHz and 0.7 W/kg of SAR. Figure 6 shows the measured temperature increase during 2 h exposure time. As evident from the temperature trend shown in Figure 7, the arrangement with two fans placed on the top and bottom of the shielding cage (see Figure 6b) guarantees that the temperature increase at the steady state is equal to 0.25 °C, which is well below the threshold for the onset of thermal effects [67].

### 4.3. Cell Cultures and Exposure Protocol

Human adult fibroblasts (HDF) (Cell Applications, Inc, San Diego, CA, USA) primary cells derived from normal human dermis were used in these experiments. Two lots of different donors were used, one derived from the normal facial skin of a 75 year-old Caucasian man (lot A) and the other one from the normal breast skin from a 54 year-old Caucasian female (lot B). Cells were cultured in Dulbecco’s Modified Eagle Medium (DMEM) (Euroclone, Pero, Italy), supplemented with 10% foetal bovine serum (Euroclone), 1% 2 mM Lglutamine, 1% penicillin/streptomycin (Thermo Fisher Scientific, Monza, Italy), and 1% non-essential amino acids (Euroclone). Cell cultures were grown incubated at 37 °C at 5% CO_2_. In order to prevent any problem of senescence or drift of the cellular population and to ensure the reproducibility of the results, all the experiments were performed on primary fibroblasts at the same passage (number 6). Cells from lot A were used for CW exposures and from lot B for PW exposures.

About 24 h before exposure, cells were seeded into 3.5 cm diameter polystyrene Petri dishes (Corning Incorporated, Corning, NY, USA) in 2 mL of medium at the density of 2 × 10^5^ cells, and the medium was changed just before exposure. The considered exposure duration was a short-term 2 h exposure. To maintain cells under appropriate conditions (37 °C, 5% CO_2_), all the exposures were performed in the incubator for cell culture. For each exposed sample, a corresponding sham-exposed control sample, kept in the same incubator without RF transmission, was included. Cells were harvested at different time points after exposure, depending on the endpoint evaluated. An independent RF exposure experiment consisted of four exposed and four sham-exposed Petri dishes. At least three independent biological replicates for each endpoint and condition and a blind evaluation of the different time-points were performed in order to minimize the individual biases.

### 4.4. Cell Cycle Analysis

The exposure effect on the cell cycle was determined by flow cytometric analysis. At and 24 h after irradiation, adherent and suspended cells were harvested, centrifuged at 1500 rpm for 10 min, and washed twice with cold phosphate buffered saline (PBS). The assay was then performed, as previously described in Benvenuto et al. [92]. Cells were analyzed with flow cytometry using a FACS Calibur cytometer, running CellQuest Pro 5.2 software (BD Biosciences, San Jose, CA, USA).

### 4.5. γ-H2AX/53BP1 Immunofluorescence Staining

Analysis of γ-H2AX/53BP1 colocalized foci was performed by immunofluorescence staining 30 min, 2 h, and 24 h after exposure. After irradiation, cells were spotted on coverslips and fixed using 2% formaldehyde/PBS for 5 min, permeabilized using 0.5% Triton-X/PBS for 5 min, and blocked using 1% bovine serum albumin (BSA) (Sigma-Aldrich, Italy) in PBS for 10 min. Cells were then incubated with a combination of 1:500 mouse monoclonal anti-γH2AX antibody (Merk Millipore Sigma-Aldrich,) and 1:1000 rabbit polyclonal anti-53BP1 antibody (Calbiochem, Sigma-Aldrich) in 1% BSA/PBS for 45 min at room temperature in a wet chamber. Subsequently, cells were washed in 1% BSA/PBS three times for 3 min and incubated in 1:500 anti-mouse Alexa Fluor 488 conjugated antibody (Molecular Probes, Life technologies, Thermo Fisher Scientific) and 1:500 anti-rabbit Alexa Fluor 555 Goat anti Rabbit IgG (Molecular Probes, Life technologies, Thermo Fisher Scientific) for 30 min at room temperature in a wet chamber in the dark. The cells were extensively washed with PBS and dried, and finally slides were mounted with 4,6-diamidino-2 phenylindole (DAPI) in Vectashield (Vector Laboratories, DBA Italia s.r.l., Italy) solution and turned upside down on the slide, and the edges were sealed using nail polish. 

Slides were viewed with an epifluorescence microscope (Imager Z1, Carl Zeiss, Germany), equipped with a charge-coupled device (CCD) camera. The automated image acquisition was performed using Metafer 4 software (version 3.6.9, from MetaSystems, MA, USA). A total of 100–250 fields of each spot were selected and acquired by the Metafer Autocapt module, using an immersion plan Apochromat oil 63X objective (Carl Zeiss). To compile all of the three-dimensionally distributed gamma-H2AX foci throughout the nuclei in one image, 26 2D-images for each field were acquired with a 0.3 µm *z*-axis step between two slides. The resulting fields of view (FOV) were transformed into training images (TNR) with the “Create TRN from FOV” to allow each color channel to be exported as an individual greyscale tiff file.

The foci scoring was performed in uncompressed high-quality images using the free cell image analysis software CellProfiler version 2.0, (Broad Institute, Cambridge, MA, USA), as described by Carpenter et al. [93].

### 4.6. Micronuclei Anti-Kinetochore Antibody (CREST) Analysis

Cytokinesis-blocked binucleated (BN) cell preparations were obtained according to the Cytokinesis Block MicroNucleus (CBMN) technique. After exposure, cytochalasin B (3 µg/mL final) (Sigma-Aldrich), was added to irradiated and sham cultures to block cytokinesis. After 24 h incubation at 37 °C, cells were harvested, treated with hypotonic solution (KCl 0.075 M), fixed in absolute ice-cold methanol, and processed for anti-kinetochore staining. Cells were washed for 3 min in PBS-Tween20 (0.01%) and in PMN solution (20% phosphate buffer pH 8, 0.5% Nonidet, 0.02% sodium azite, 5% fat-free milk powder and H_2_O). CREST anti-kinetochore antibody (Antibody Inc. Davis, CA, USA) 1:1 in PBS-Tween20 (0.1%) was put on slides that were incubated overnight in a wet chamber at 37 °C. Three wash steps were then performed in PBS/BSA 1% (5 min) and one in PMN. The Rabbit anti-human IgA, IgG, IgM (H + L), FITC-conjugated secondary antibody (Sigma Immunochemicals, St. Louise, MO, USA) was then added on the slides in a 1:80 dilution in PBS/BSA 1%. Slides were let dry for 45 min in a wet chamber at 37 °C. Three washing steps in PBS/BSA 1% were performed, followed by one wash in cold PBS 1X. Slides were then counterstained with DAPI (2 µg/mL) 1:1 with Vechashield antifade. MN were classified for the presence (CREST-positive, MN+) or absence (CREST-negative, MN-) of kinetochore reaction under appropriate filters for DAPI and FITC, using an 40× objective. The scoring was performed using a fluorescence microscope Axio Imager A1 (Carl Zeiss, Berlin, Germany), following the scoring criteria described for MN in Fenech and Morley [19].

### 4.7. Statistical Analysis

Statistical analyses have been performed using different tests, according to the assay. A *t*-test was used for γH2AX/53BP1 and FACS analyses. The chi-squared test was carried out for MN-CREST analyses. Statistical significance was considered for the value *p* < 0.05.

### 4.8. Transmission Electron Microscopy

Ultrastructural analysis was performed on samples 2 h and 24 h after exposure by cell observation with transmission electron microscopy. Cells were fixed in 2.5% glutaraldehyde in PBS (pH 7.4) at 4 °C and post-fixed with 1.33% osmium tetroxide, dehydrated in graded alcohols, and then embedded in Epon 812 resin (Fisher Chemical Co., Dallas, TX, USA). The resin was allowed to polymerize in a dry oven at 60 °C, and specimens were cut on a Reichert-Jung ultra-microtome (Leica Microsystems GmbH, Wetzlar, Germany), stained with uranyl acetate and lead citrate, and observed under a Philips Morgagni 268D transmission electron microscope (Thermo Fisher Scientific, Waltham, MA, USA) [94]. 

### 4.9. Gene Expression Profiling

#### 4.9.1. RNA Extraction 

Total RNA from exposed samples and the respective control sham samples, was isolated using QIAamp RNA Blood Mini Kit (Qiagen) immediately after, 2 h after, and 24 h after 2.45 GHz (CW or PW) exposure, according to the manufacturer’s instructions. RNA concentration was measured using QuantiFluor RNA system (Promega, Madison, WI, USA) and the samples were stored at −80 °C.

#### 4.9.2. mRNA Sequencing

For the sample preparation, TruSeq Stranded mRNA (Illumina, San Diego, CA, USA) was used and the starting amount of total RNA was of 500 ng/µL. From the total RNA, poly-A mRNA was purified using poly-T oligo attached magnetic beads. First and second strand cDNA was synthesized obtaining blunt-ended cDNA and a single ‘A’ nucleotide was added to the 3′ ends of the blunt fragments. The adapter oligonucleotides were ligated to the cDNA and amplified by a PCR, performed with a PCR Primer Cocktail (Illumina) that anneals to the ends of the adapters. Prior to sequencing, the libraries were validated, checking the size (approximately 260 bp) and purity using a DNA-specific chip (Agilent DNA 1000) on an Agilent Technologies 2100 Bioanalyzer (Santa Clara, CA, USA). After validation, the libraries were normalized and pooled. The pool to be sequenced was then denatured and diluted in the resulted optimal concentration (1.2 pM). High-throughput next generation sequencing was performed using the Illumina sequencing technology platform (NexSeq500). The output of the sequencing consists of generating raw reads (FASTQ format) as starting material for the analysis of the mRNA-seq data.

#### 4.9.3. Data Analysis and Statistical Methodology

Reads generated during the Illumina sequencing process were quality checked using FastQC (v.0.11.7) (Babraham Institute, Cambridge, UK). Trimming of the poor-quality bases (qscore < 20) was performed using the Sickle software (v.1.33) (UC Davis Bioinformatics Core, Davis, CA, USA) (https://github.com/najoshi/sickle). The trimmed reads were mapped to the human reference genome (UCSC hg19 version) using the Subread software (v.1.6.1) (Walter and Eliza Hall Institute of Medical Research, Parkville, Victoria, Australia) with default parameters. The number of reads, trimmed reads, and aligned reads for each sample are reported in Supplementary Table S1. At gene level, the reads quantification was performed with the featureCounts tool (v.1.6.1), with the human genome annotation file in GTF format. In order to identify the differentially expressed genes (DEGs), the DESeq2 (v.1.28.1) and EdgeR (v.3.30.3) R packages were applied. A false discovery rate (FDR) with *p*-adjusted ≤ 0.01, was used as parameter to identify differentially expressed genes. Results of the statistical analysis without FDR adjustment, considering a *p*-value of ≤ 0.05 and fold change of 1.5 as the minimum cut-off value, were also considered. Heat maps were used to display the expression profile of genes differentially expressed at different time points in CW and PW conditions (Supplementary Figure S1).

#### 4.9.4. Functional Analysis of Differentially Expressed Genes

To perform the characterization of molecular functions or pathways, in which DGEs are involved, the Protein Analysis Through Evolutionary Relationships (PANTHER) classification system was used [95]. This classification system is part of the Gene Ontology (GO) Phylogenetic Annotation Project, and the analysis of the screened DGEs was performed on the three ontology levels, biological processes, molecular functions, and pathways.

#### 4.9.5. Validation Experiments Using Real-Time Quantitative PCR (qRT-PCR)

Based on mRNA-seq results, five candidate transcripts were selected based on the higher expression values in comparison to sham control samples, for validation by qRT-PCR.

Aliquots of total RNA (1 µg) were reverse-transcribed (1×/25 °C/10 min, 1×/37°C/ 120 min, 1 × 85 °C/5 min, 1 × 8 °C/10 min) with the High-Capacity cDNA Reverse Transcription Kit (Applied Biosystem, CA, USA).

The PCR reaction was performed in duplex and contained TaqMan Universal PCR Master Mix (Applied Biosystem), 18S rRNA as the endogenous control (Applied Biosystem), and one of five inventoried TaqMan minor groove binder assays for detection of candidate transcripts, using commercially available inventoried primer probe designs, listed in Table 5. The qRT-PCR run was performed on the StepOne (AB) platform (1×/50 °C/2 min 1×/95 °C/10 min 40×/95 °C/1 min 60 °C/1 min).

The cycle threshold (Ct) values of the genes of interest were normalized, relative to 18S rRNA. On each plate, we added a no template control, and PCRs were performed in triplicate on four cDNA preparations, corresponding to four independent exposures. The 18S rRNA-Ct values were used as quality control markers for the cDNA synthesis. The relative expression of each mRNA was calculated by the ΔC_t_ method, and data were expressed as fold of induction, which correspond to the ratio of exposed mRNA to sham mRNA.

## Figures and Tables

**Table 1:** Cell cycle analysis on HDF cells exposed to 2.45 GHz CW and PW. No differences were observed 2 and 24 h after irradiation with both continuous wave (CW) and pulsed wave (PW) signals. Results are representative of three experiments. Results obtained with the cells exposed to CW or PW signals were compared to those obtained with the corresponding sham cells (exposed 2 h vs. sham 2 h; exposed 24 h vs. sham 24 h; 2-tailed *t*-test). Not significant (NS).

2.45 GHz	Time Point	Sample	Sub-G1 ^1^		G0/G1		S		G2/M	
			Mean	*p*	Mean	*p*	Mean	*p*	Mean	*P*
CW	2 h	Sham	1.92 ± 0.42		50.92 ± 12.97		6.85 ± 1.77		40.61 ± 11.66	
		Exposed	1.44 ± 0.76	NS	50.12 ± 12.95	NS	7.97 ± 2.18	NS	40.81 ± 10.13	NS
	24 h	Sham	0.80 ± 0.66		86.03 ± 2.33		3.46 ± 0.49		9.85 ± 2.35	
		Exposed	0.82 ± 0.70	NS	86.31 ± 2.61	NS	3.53 ± 0.59	NS	9.50 ± 2.81	NS
PW	2 h	Sham	0.71 ± 0.13		72.84 ± 1.12		4.06 ± 0.61		22.62 ± 1.58	
		Exposed	0.84 ± 0.13	NS	75.00 ± 0.97	NS	4.17 ± 0.51	NS	20.23 ± 1.43	NS
	24 h	Sham	0.49 ± 0.16		84.10 ± 0.54		2.00 ± 0.17		13.53 ± 0.41	
		Exposed	0.44 ± 0.18	NS	82.43 ± 1.47	NS	2.24 ± 0.34	NS	15.03 ± 1.02	NS

^1^ Percentage of cells in the sub-G1, G0/G1, S and G2/M phase were calculated using Cell Quest software.

**Table 2:** List of genes differentially expressed between exposed and sham samples after exposure to 2.45 GHz CW. The table includes gene name, gene function, fold change (FC), and regulation.

2.45 GHz CW	Gene ID	Gene Name	Description	FC	Regulation
0 min after exposure	ACOT4	Acyl-coenzyme A thioesterase 4	Signaling receptor binding and palmitoyl-CoA hydrolase activity	1.6	up
	DLX5	Homeobox protein DLX-5	Transcriptional factor involved in bone development	2	up
	FAM72D	Protein FAM72D	Unknown function	2	up
	LRP2BP	LRP2-binding protein	Protein binding	2.5	down
	NIPSNAP3B	Protein NipSnap homolog 3B	Rutative roles in vesicular trafficking	2.2	up
	SUN3	SUN domain-containing protein 3	Protein binding	2.3	up
2 h after exposure	HES4	Transcription factor HES-4	Basic helix-loop-helix transcription factor	1.6	down
	HMCN1	Hemicentin-1	Receptor binding	1.8	up
	ALMS1	Alstrom syndrome protein 1	Cytoskeletal protein binding	1.5	up
	ANKRD36C	Ankyrin repeat domain-containing protein 36	Protein Coding gene involved in ion channel inhibitor activity	1.6	down
	GRIP2	Glutamate receptor-interacting protein 2	Multi-PDZ domain scaffolding proteins required for dendrite development	1.5	down
	BSN	Protein bassoon	Scaffolding protein involved in organizing the presynaptic cytoskeleton	1.6	up
	FAM53A	Protein FAM53A	Encodes a secreted peptide hormone and member of the EGF family of proteins	1.5	down
	EREG	Epiregulina	Transient receptor potential cation channel	1.5	down
	SLC9B1	Sodium/hydrogen exchanger 9B1	Transmembrane transporter activity	1.7	down
	LUCAT1	lung cancer associated transcript 1	Non-coding RNA	1.6	down
	EGR1	Early growth response protein 1	Transcriptional regulator	1.7	up
	AC005618.6	Protocadherin gamma-B3	Cell adhesion, cell-cell signaling	1.8	up
	HIST1H2AD	Histone H2A type 1-D	Histone	2	down
	HIST1H2BG	Histone H2B type 1-C/E/F/G/I	Histone	1.8	down
	IER3	Radiation-inducible immediate-early gene IEX-1	Cell proliferation and survival	2.2	down
	PRSS35	Inactive serine protease 35	Serin protease activity	1.7	down
	SAMD3	Sterile alpha motif domain-containing protein 3	Protein binding	1.8	up
	GPER1	G-protein coupled estrogen receptor 1	Protein binding	1.5	down
	RIMS2	Regulating synaptic membrane exocytosis protein 2	Protein binding	1.6	up
	ZNF462	Zinc finger protein 462	Protein binding	1.7	up
	SYNPO2L	Synaptopodin 2-like protein	Protein binding	1.8	up
	CNNM1	Metal transporter CNNM1	Protein binding	1.5	down
	C11orf96	Uncharacterized protein C11orf96		1.7	down
	BEST1	Bestrophin-1	Ion binding	1.6	up
	STYK1	Tyrosine-protein kinase STYK1	Receptor binding	1.5	up
	RPPH1	Ribonuclease P RNA Component H1	long non-coding RNA	3.9	down
	RN7SL1	RNA, 7SL, cytoplasmic 1	small cytoplasmic RNA	2.7	down
	RN7SL2	RN7SL2	small cytoplasmic RNA	4.1	down
	RHOJ	Rho-related GTP-binding protein RhoJ	small GTPase	1.5	down
	SYNE2	Nesprin-2	Actin binding	2	up
	AHNAK2	Protein AHNAK2	RNA binding	1.5	up
	FBXL22	F-box and leucine-rich protein 22	Protein ubiquitination	1.6	up
	SLC43A2	Large neutral amino acids transporter small subunit 4	Transmembrane transporter activity	1.7	up
	MYCBPAP	MYCBP-associated protein	Cell differenziation	2	up
	ZNF433	Zinc finger protein 433	DNA-binding (transcription)	1.3	up
	ZNF233	Zinc finger protein 233	Nucleic acid binding, regulation of transcription	1.8	up
	RP1-198K11.5	Non-coding RNA	1.5	down
	MXRA5	Matrix-remodeling-associated protein 5	Receptor binding	1.8	up
	RMRP	RNA Component Of Mitochondrial RNA Processing Endoribonuclease	Non-coding RNA	8.8	down
24 h after exposure	ANKRD36C	Ankyrin repeat domain-containing protein 36	Protein Coding gene, ion channel inhibitor activity	1.6	down
	MIR145	microRNA 145	Non-coding RNA	1.4	up
	KIAA0895	Uncharacterized protein KIAA0895		1.8	up
	LCNL1	Lipocalin-like 1 protein	Binding, isomerase activity	2.3	up
	RASGEF1A	Ras-GEF domain-containing family member 1A	protein binding, small GTPase regulator activity	1.5	up
	RN7SL1	RNA, 7SL, cytoplasmic 1	small cytoplasmic RNA	1.8	down
	RN7SL2	RNA, 7SL, cytoplasmic 2	small cytoplasmic RNA	1.8	down
	RGS11	Regulator of G-protein signaling 11	Regulator of G protein signaling	1.7	up

**Table 3:** List of genes differentially expressed between exposed and sham samples after exposure to 2.45 GHz PW. The table includes gene name, gene function, fold change (FC), and regulation.

2.45 GHz PW	Gene ID	Gene Name	Description	FC	Regulation
0 min after exposure	KIAA1324	UPF0577 protein KIAA1324	RNA binding	1.6	up
	KIAA1211	Uncharacterized protein KIAA1211	Unknown function	2.3	up
	CXCL3	C-X-C motif chemokine 3	Chemokine	1.6	down
	EGR3	Early growth response protein 3	Transcriptional regulator	1.5	down
	SLC16A13	Monocarboxylate transporter 13	Transmembrane transporter	1.5	up
2 h after exposure	TMEM240	Transmembrane protein 240	Transmembrane-domain containing protein	1.54	up
	TNFRSF25	Tumor necrosis factor receptor superfamily member 25	Signaling receptor activity	1.7	up
	BEST4	Bestrophin-4	Anion channel	1.8	up
	RNF175	RING finger protein 175	Ubiquitin- protein ligase	1.7	up
	KLKB1	Plasma kallikrein	Serin-protease	1.5	down
	PTGER4	Prostaglandin E2 receptor EP4 subtype	G-protein coupled receptor	2	down
	MDFI	MyoD family inhibitor	Transcription factor binding	2	up
	PPP1R9A	Neurabin-1	Actin binding	1.9	down
	RMRP	RNA Component Of Mitochondrial RNA Processing Endoribonuclease	Non-coding RNA	5.7	down
	PRUNE2	Protein prune homolog 2	Pyrophosphatase activity	1.6	down
	ENO4	Enolase 4	Lyase activity	1.9	down
	KCNQ1OT1	KCNQ1 opposite strand/antisense transcript 1	non-coding RNA	1.7	down
	OLR1	Oxidized low-density lipoprotein receptor 1	Lipoprotein receptor	2.1	down
	HOXC11	Homeobox protein Hox-C11	Transcription factor	1.7	up
	PTPRQ	Receptor-type tyrosine-protein phosphatase R	Protein phosphatase	1.8	down
	PAPLN	Papilin	Peptidase activity	1.7	down
	TMEM121	Transmembrane protein 121		2	up
	ATF7IP2	Activating transcription factor 7-interacting protein 2	ATPase activity	2.4	down
	CNBD2	Cyclic nucleotide-binding domain-containing protein 2	cAMP binding	1.7	up
	AHNAK2	Protein AHNAK2	RNA binding	1.8	down
	LIF	Leukemia inhibitory factor	Cytokine activity	1.8	down
	PDZD4	PDZ domain-containing protein 4	Ubiquitin protein ligase activity	2.1	up
24 h after exposure	PPP1R1C	Protein phosphatase 1 regulatory subunit 1C	Signaling molecule, phosphatase inhibitor	2.6	up
	ADAMTS13	A disintegrin and metalloproteinase with thrombospondin motifs 13	Metallopeptidase activity	1.5	up
	PANO	Proapoptotic Nucleolar Protein 1	Apoptosis-inducing protein	1.9	up
	NEAT1	nuclear paraspeckle assembly transcript 1	Non-coding RNA	1.5	up
	VAMP1	Vesicle-associated membrane protein 1	Transport	1.5	up
	GOLGA8B	Golgin subfamily A member 8B	Membrane traffic protein	1.5	up
	GUSBP11	Putative inactive beta-glucuronidase protein GUSBP11	Hydrolase activity	1.5	up

**Table 4:** In the table, the results obtained from the RT-PCR validation on the selected genes, compared to the results obtained from RNA-seq analysis are reported. The validation was performed on the same RNA used for RNA-seq, and the results are representative of four independent experiments. These genes were differentially expressed 2 h after exposure after CW or PW signals, and results obtained with the exposed cells were compared to the corresponding sham cells (exposed 2 h vs. sham 2 h; *t*-test). The table includes type of signal, gene name, fold change (FC), direction of change as up-regulation (↑), down-regulation (↓) or no changes (-) and *p*-value. Not significant (NS).

2.45 GHz	Gene	RNA-seq			RT-PCR		
		FC	Regulation	*p*-Value	FC	Regulation	*p*-Value
CW	RMRP	8.8	↓	≤0.05	0.73	↓	NS
	ALMS1	2.06	↑	≤0.05	1.14	↑	NS
	BSN	2.37	↑	≤0.05	1.52	↑	≤0.001
	SYNE2	2.06	↑	≤0.05	2.1	↑	NS
	AHNAK2	2.18	↑	≤0.05	1.0	-	NS
PW	RMRP	5.7	↓	≤0.05	0.96	↓	NS
	AHNAK2	1.5	↓	≤0.05	1.99	↑	NS

**Table 5:** Candidate transcripts selected for the validation by qRT-PCR. Inventoried primer probe designs were used.

Gene Name	Description	Assay ID
RMRP	RNA Component of Mitochondrial RNA Processing Endoribonuclease	Hs03298751_s1
ALMS1	Cytoskeletal protein binding	Hs00367316_m1
BSN	Scaffolding protein involved in organizing the presynaptic cytoskeleton	Hs01109152_m1
SYNE2	Actin binding	Hs00794881_m1
AHNAK2	RNA binding	Hs00292832_m1

## Supplementary Materials

The following are available online at https://www.mdpi.com/1422-0067/21/19/7069/s1.
