# Supplementary figures and images for: Human Fibroblasts In Vitro Exposed to 2.45 GHz Continuous and Pulsed Wave Signals: Evaluation of Biological Effects with a Multimethodological Approach
Source: Int J Mol Sci. 2020 Sep 25;21(19):7069. doi: 10.3390/ijms21197069 (PMC7584027; doi:10.3390/ijms21197069)

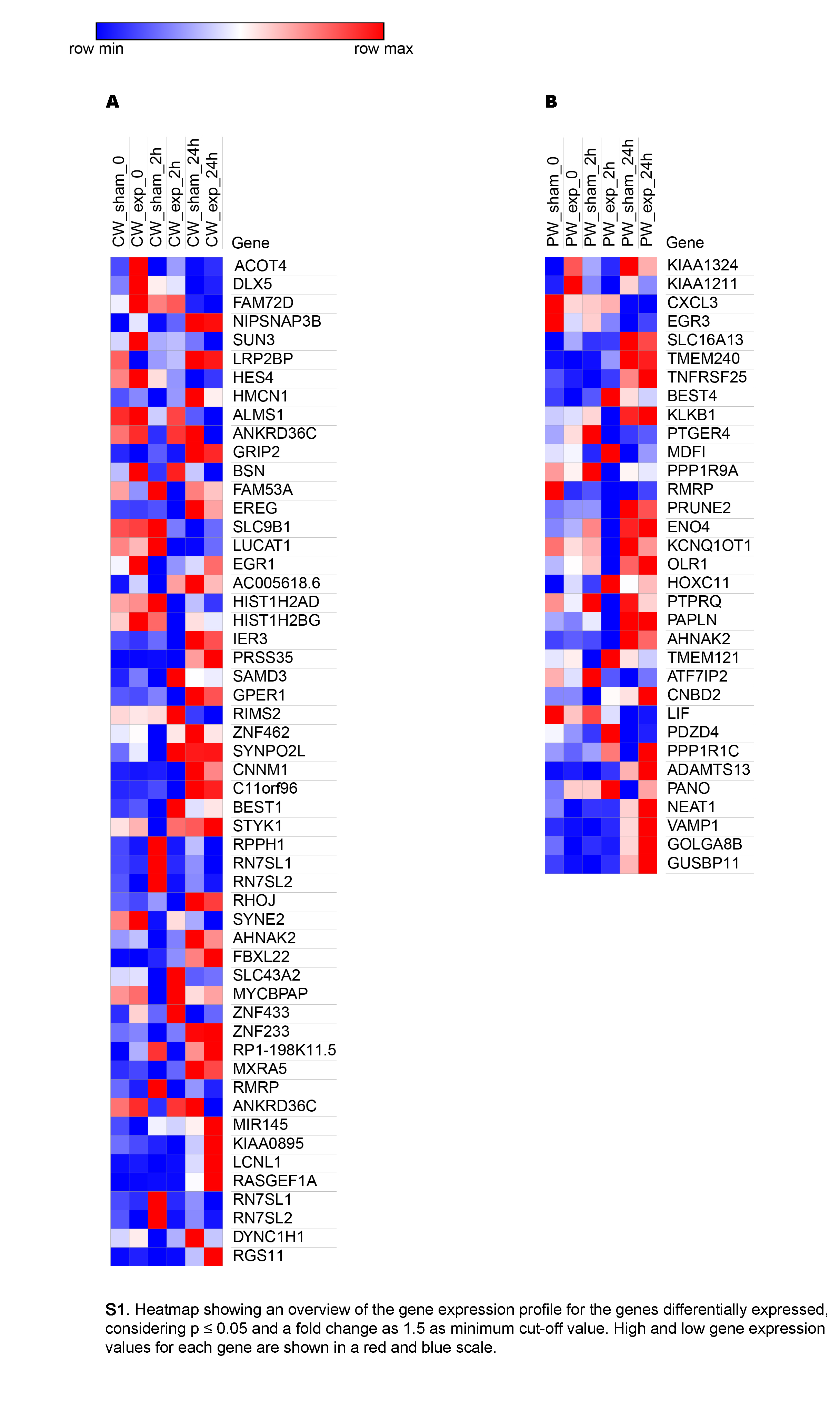

Supplement: Supplementary file 1 [file ijms-21-07069-s001.zip › ijms-905074 sup/Supplementary material/Fig. S1.jpg]
