# Supplementary material for: Human Fibroblasts In Vitro Exposed to 2.45 GHz Continuous and Pulsed Wave Signals: Evaluation of Biological Effects with a Multimethodological Approach
Source: Int J Mol Sci. 2020 Sep 25;21(19):7069. doi: 10.3390/ijms21197069 (PMC7584027; doi:10.3390/ijms21197069)
Supplement: Supplementary file 1 [file ijms-21-07069-s001.zip › ijms-905074 sup/Supplementary material/Tab S1.doc]

| **Type of signal** | **Time point** | **Sample** | **Tot reads** | **Trimmed reads** | **Mapped reads** |
| --- | --- | --- | --- | --- | --- |
| 2.45 CW | 0 min after exposure | sham | 54 102 714 | 51 552 654 | 45 713 923 |
|  |  | exp | 53 273 137 | 55 153 520 | 45 512 987 |
|  | 2 h after exposure | sham | 51 806 110 | 49 362 325 | 43 252 900 |
|  |  | exp | 54 044 357 | 51 237 238 | 45 076 357 |
|  | 24 h after exposure | sham | 59 505 760 | 56 548 261 | 49 521 033 |
|  |  | exp | 59 192 365 | 56 587 843 | 49 743 128 |
| 2.45 PW | 0 min after exposure | sham | 55 648 438 | 51 957 987 | 47 122 862 |
|  |  | exp | 56 529 371 | 52 816 263 | 49 671 254 |
|  | 2 h after exposure | sham | 51 380 658 | 48 362 104 | 45 367 451 |
|  |  | exp | 51 380 771 | 47 956 816 | 45 052 927 |
|  | 24 h after exposure | sham | 46 487 096 | 43 052 641 | 40 335 111 |
|  |  | exp | 56 316 666 | 52 265 951 | 48 772 833 |

**Table S1.** In the table are reported the number of reads, trimmed reads and aligned reads. The data are representative of four experiments replicates for each time point and condition evaluated.
